# Dermatological manifestations in patients with gastrointestinal malignancies: a focus on early diagnosis and patient comfort^☆^

**DOI:** 10.1016/j.abd.2025.501128

**Published:** 2025-06-18

**Authors:** Burcu Aydemir Demirci, Deniz Aksu Arica, Leyla Baykal Selcuk, Evren Fidan, Ali Guner, Savas Yayli, İbrahim Etem Arica

**Affiliations:** a Department of Dermatology and Venereology, Zonguldak Ataturk City Hospital, Zonguldak, Turkey; b Department of Dermatology and Venereology, Karadeniz Technical University, Faculty of Medicine, Trabzon, Turkey; c Department of Medical Oncology, Karadeniz Technical University, Faculty of Medicine, Trabzon, Turkey; d Department of General Surgery, Karadeniz Technical University, Faculty of Medicine, Trabzon, Turkey; e Department of Dermatology and Venereology, Koç University, Faculty of Medicine, Istanbul, Turkey

**Keywords:** Acanthosis nigricans, Dermatology, Gastrointestinal neoplasms, Keratosis, Seborrheic, Paraneoplastic syndromes

## Abstract

**Background:**

The increasing prevalence of gastrointestinal (GI) malignancies highlights the critical need for a better understanding of associated dermatological manifestations to improve early diagnosis and patient management.

**Objectives:**

This study aims to catalog the dermatological findings in patients with GI malignancies, emphasizing the implications for early diagnosis, and management.

**Methods:**

The authors conducted a comprehensive whole-body dermatologic examination on 150 patients diagnosed with GI malignancies. Data on sociodemographic factors, lifestyle habits, comorbid conditions, and malignancy characteristics were meticulously collected.

**Results:**

The cohort comprised 96 males (64%) and 54 females (36%), with a mean age of 62.47 (SD ± 10.7) years. The most prevalent primary malignancy was colonic (32.7%), with a mean disease duration of 12.72 (SD ± 20.5) months. Xerosis (dry skin) was the most frequent dermatological condition, affecting 92% of patients. The itching was reported by 31.3% of patients. Eruptive seborrheic keratosis (20.7%) and acanthosis nigricans (10.7%) were the most common paraneoplastic dermatoses observed. Both conditions were more common in patients with BMIs above 28.5, and seborrheic keratosis was seen more often in men. Dry skin, itching, paraneoplastic dermatoses, and skin infections were significantly more prevalent in patients older than the mean age of 62 years.

**Study limitations:**

The cross-sectional design of the present study limits causal inference. Longitudinal studies are needed to explore the temporal dynamics between dermatological manifestations and GI malignancy progression.

**Conclusions:**

Dermatologic symptoms including pruritus, xerosis, seborrheic keratosis, and acanthosis nigricans should be considered potential indicators of underlying malignancy. Routine dermatological screening is essential for comprehensive patient care.

## Introduction

The global incidence of gastrointestinal (GI) malignancies is on the rise, presenting increasing challenges to healthcare systems worldwide.^1^ Patients suffering from these malignancies constitute a particularly vulnerable group, facing not only the primary effects of the cancer and its systemic repercussions but also the adverse effects associated with various treatment modalities. Dermatological manifestations are emerging as significant both for their potential in early cancer detection and for their influence on patient quality of life.^2^, ^3^

Dermatological complications in these patients may include paraneoplastic skin conditions, which could act as early indicators of underlying malignancy, as well as a spectrum of skin disorders that are often exacerbated by immunosuppression and adverse reactions to medications.^3^, ^4^, ^5^, ^6^ The increased prevalence of skin conditions in this patient population offers dermatologists a crucial role in the multidisciplinary approach to managing individuals with GI malignancies, highlighting the importance of dermatological assessment in this context.

This study explores dermatological manifestations in patients with gastrointestinal (GI) malignancies with two main objectives: enhancing early cancer detection and improving management of skin conditions. By examining these correlations, the authors aim to advance early diagnosis and comprehensive treatment of GI cancers, thereby improving patient outcomes and quality of life.

## Methods

This cross-sectional study was conducted in collaboration with the Departments of Dermatology, Medical Oncology, and General Surgery at the Faculty of Medicine, Karadeniz Technical University. The study included a cohort of patients who presented to the respective outpatient clinics over a one-year period from April 2021 to April 2022.

A total of 150 patients with a confirmed clinical and histopathological diagnosis of gastrointestinal system malignancy were enrolled in the study. Inclusion criteria were a definitive diagnosis of GI malignancy, while exclusion criteria included patients without a confirmed histopathological diagnosis and those who declined to participate in the study.

Sociodemographic data (age, sex, occupation, education level), lifestyle habits (smoking status, alcohol consumption), comorbidities, and detailed information related to the primary malignancy (type, stage, duration of illness) were meticulously recorded for each patient. A standardized data collection form was utilized to ensure the consistency and reliability of the gathered information. All participants underwent a comprehensive whole-body dermatological examination upon their initial visit. The examination was performed by experienced dermatologists in accordance with established protocols to identify both general dermatological conditions and specific paraneoplastic skin findings. The dermatological findings were meticulously documented using a standardized reporting form to facilitate subsequent data analysis.

The study was approved by the Institutional Review Board (IRB) of Karadeniz Technical University Faculty of Medicine. All procedures performed in the study adhered to the ethical standards of the institutional and/or national research committee and with the 1964 Helsinki Declaration and its later amendments or comparable ethical standards. Informed consent was obtained from all individual participants included in the study.

### Statistical methods

Data analysis was conducted using the SPSS statistical software package, version 23.0. During the statistical evaluation, quantitative data were expressed as mean ± Standard Deviation (SD), while categorical data were presented as frequencies and percentages (%). The normality of distribution for the quantitative data was assessed using the Kolmogorov-Smirnov test. For data following a normal distribution, comparisons between two means were performed using the Student's *t*-test or the Z-test, as appropriate. The Mann-Whitney *U* test was employed for comparisons of data not conforming to a normal distribution. Categorical data were compared using the Chi-Square test. Correlations between parameters were examined using Pearson's or Spearman's correlation analysis depending on the distribution type. The level of significance was set at a p-value of less than 0.05 (p < 0.05).

## Results

In this study, the authors included a total of 150 patients diagnosed with GI malignancies, of which 96 (64%) were male, and 54 (36%) were female. The mean age was 62.47 years with a standard deviation of 10.7 years. The Fitzpatrick skin types of the patients varied, with type 3 being the most common at 42.7%, followed by type 4 (29.3%), type 2 (26.7%), and type 5 (1.3%). The average Body Mass Index (BMI) was 25.86 with a standard deviation of 5.7.

The primary malignancies observed were predominantly colon (32.7%), stomach (32%), and rectum (16.7%). Adenocarcinoma was the most frequent histological subtype, accounting for 93.3% of cases. The mean duration of the disease was 12.72 months (±20.5 months), calculated from the initial diagnosis to the inclusion in the study. A majority of the patients (80.7%) presented with advanced disease (stages 3 and 4), and 56.7% were undergoing chemotherapy at the time of evaluation. Sociodemographic and clinical characteristics are summarized in Table 1.

**Table 1:** The sociodemographic and clinical characteristics of the study population.

Age, mean (±)	62.47 (±10.7)
Sex, n(%)	
Male	96 (64%)
Female	54 (36%)
Body Mass Index (BMI) (±SD)	25.86 (±5.7)
Habits, n (%)	
Smoker	11 (7.3%)
Alcohol consumption	2 (1.3%)
Comorbidities, n (%)	
Hypertension	58 (38.7%)
Diabetes mellitus	33 (22%)
Cardiovascular disease	38 (25.3%)
Hyperlipidemia	23 (15.3%)
Malignancy subtype, n (%)	
Oral cavity	1 (0.7%)
Esophagus	5 (3.3%)
Stomach	48 (32%)
Small intestine	1 (0.7%)
Colon	49 (32.7%)
Rectum	25 (16.7%)
Pancreas	14 (9.3%)
Gallbladder	2 (1.3%)
Liver	5 (3.3%)
Disease duration, (mean ± SD), month	12.72 (±20.5)

The most prevalent dermatological condition observed in this study was xerosis (dry skin), affecting 92% of the patients. A higher incidence of dry skin was noted particularly in patients older than the mean age of 62 years, and this finding was statistically significant (p = 0.014). Pruritus (itching) was reported by 31.3% of the cohort, occurring more frequently among those diagnosed with colorectal and gastric adenocarcinomas. However, when comparing the incidence of pruritus across different types of malignancies, no statistically significant difference was found. Additionally, pruritus was more commonly experienced by patients above the mean age, with this correlation reaching statistical significance (p = 0.007).

Eruptive seborrheic keratosis (20.7%) and acanthosis nigricans (10.7%) were the most frequent paraneoplastic skin manifestations. Both conditions were more common in patients with Body Mass Index (BMI) values above 28.5 (p = 0.04 and p < 0.001, respectively), and seborrheic keratosis was seen more often in men and those over the age of 62 (p = 0.01 and p = 0.003, respectively). 37.5% of patients with acanthosis nigricans had concurrent diabetes.

The distribution of seborrheic keratosis and acanthosis nigricans among the patients was characterized by their anatomical location and frequency. Seborrheic keratosis was predominantly located on the trunk (48.4% of patients), followed by the head and neck region (41.9%). A total of 45.2% of patients exhibited between 50 and 100 lesions. Acanthosis nigricans showed a predilection for the inguinal area, with 35.3% of affected patients reporting it in this region. The neck was the next most common site (29.4%), followed by the axilla (23.5%). In some cases, patients showed manifestations in both axillary and inguinal regions (11.8%).

Acquired ichthyosis was found in 5.3% of the patient cohort, with a temporal association with malignancy diagnosis. Among these patients, stomach adenocarcinoma was the most common underlying malignancy (five patients), followed by esophageal squamous cell carcinoma, colon adenocarcinoma, and pancreatic adenocarcinoma, with one patient each.

Acquired hypertrichosis lanuginosa was observed in 4% of patients, with colon adenocarcinoma being identified in 50% of these cases. Other malignancies associated with hypertrichosis lanuginosa included esophageal squamous cell carcinoma, hepatocellular carcinoma, and gastric adenocarcinoma, each comprising 16.7% of cases. This condition was significantly more frequent in female patients (p = 0.023).

Table 2 details the statistical analysis of dermatological findings in relation to age, sex, BMI, and malignancy type.

**Table 2:** Statistical analysis of dermatological findings by age, gender, body mass ındex, and type of malignancy.

	Seborrheic keratosis. n (%)	p-value	Acanthosis nigricans. n (%)	p-value	Acquired ichthyosis. n (%)	p-value	Acquired hypertrichosis lanuginosa. n (%)	p-value
Age		**0.003**		0.310		0.160		0.090
<62	8 (10.8)		6 (8.1)		2 (2.7)		5 (4)	
>62	23 (30.3)		10 (13.2)		6 (7.9)		1 (1.3)	
Sex		**0.010**		0.890		0.150		**0.014**
Female	5 (9.3)		6 (11.1)		1 (1.8)		5 (9.2)	
Male	26 (27.1)		10 (10.4)		7 (7.3)		1 (1)	
Body Mass Index		**0.039**		**<0.001**		0.120		0.920
<25.8	20 (27.8)		1 (1.4)		6 (8.3)		3 (4.2)	
>25.8	11 (14.1)		15 (19.2)		2 (2.6)		3 (3.8)	
Malignancy type		0.460		0.250		0.084		0.550
Gastric	8 (16.7)		3 (6.2)		5 (10.4)		1 (2.1)	
Colorectal	15 (20.3)		11 (14.9)		1 (1.3)		3 (4.1)	
Other	8 (28.6)		2 (7.1)		2 (7.1)		2 (7.1)	

Dermatological assessment of facial conditions in this cohort indicated that rosacea was the predominant diagnosis, affecting 38.7% of the patients. Within this subset, 93.1% were classified as having the erythematotelangiectatic subtype of rosacea. The most frequently associated malignancy among patients with rosacea was colorectal adenocarcinoma, accounting for 48.3% of cases. Seborrheic dermatitis was identified in 6.7% of the patient population. Of those with seborrheic dermatitis, a significant majority (80%) were diagnosed with colorectal adenocarcinoma.

Upon examination of the patient cohort for premalignant and malignant skin neoplasms, the authors identified multiple actinic keratoses (defined as more than 10 lesions) in 3.3% of patients. In addition, Bowen's disease and basal cell carcinoma were each detected in 0.7% of the patient population.

Additional dermatological findings in our patient cohort were as follows: multiple acrochordons, with a presentation of more than ten lesions, were observed in 6% of patients. A considerable prevalence of cherry angiomas, also characterized by more than ten lesions, was noted in 21.3% of the patients. Palmar telangiectasias were identified in 4% of the cohort. Conditions with a prevalence of 2.7% included trichostasis spinulosa and keratosis pilaris. Additionally, 2% of patients were found to have decubitus ulcers, and 0.7% presented with post-zoster granulomatous dermatitis.

In terms of infectious dermatological conditions within our patient cohort, the following prevalences were observed: tinea unguium was the most common, affecting 68.7% of patients, followed closely by tinea pedis at 62%. Tinea cruris was present in 4% of patients. Oral candidiasis was diagnosed in 34.7% of patients, indicating a significant incidence of this opportunistic infection. Pityriasis versicolor was identified in 2% of the cohort, while scabies were less common, and found in 0.7% of patients. Herpes zoster was noted in 6.7% of patients; it was documented approximately two years prior to the diagnosis of malignancy in eight patients (5.3%), following the diagnosis in one patient, and one patient presented with an active infection during the examination.

The most prevalent hair-related finding was androgenetic alopecia, affecting 58.7% of the patient population. This was followed by chemotherapy-associated anagen effluvium, which was observed in 21.3% of the patients.

Within the assessment of nail disorders in our patient cohort, excluding tinea unguium, longitudinal ridging emerged as the predominant condition, affecting 20.7% of patients. Additional nail abnormalities observed included brittle nails (onychoschizia) in 9.3% of the patients, leukonychia in 6.7%, Mees' lines in 6%, Muehrcke's lines in 3.3%, splinter hemorrhages in 4%, and Beau's lines in 0.7% of the cohort.

In terms of oral mucosal health, oral candidiasis was notably present in 34.7% of patients, reflecting a significant concern for oral health in this population. Additional findings were atrophic glossitis in 7.3%, fissured tongue (lingua plicata) in 2.7%, geographic tongue (benign migratory glossitis) in 2%, black hairy tongue also in 2%, and oral aphthous ulcers in 0.7% of the patients.

When analyzing the data based on age and gender, it was observed that certain dermatological conditions were significantly more prevalent in patients older than 62 years. These conditions included paraneoplastic dermatoses, cherry angiomas, rosacea, onychomycosis, tinea pedis, and herpes zoster, with p-values of 0.008, 0.007, 0.027, 0.006, 0.003, and 0.050, respectively. Additionally, seborrheic dermatitis and tinea pedis were found to be more common in female patients, with p-values of 0.014 and 0.023, respectively.

## Discussion

The present study contributes to the growing body of literature on the association between malignancies and dermatologic manifestations, particularly in patients with GI cancers. This correlation has intrigued clinicians for decades, yet the lack of prospective epidemiological studies has hindered a comprehensive understanding. By cataloging skin findings in our cohort, the authors aimed to enhance clinical awareness and provide a reference for future research.

### Itching and malignancy

Pruritus, commonly known as itching, has been increasingly recognized as a symptom that may signal the presence of systemic diseases, including cancer.^7^ A substantial study by Larson Valerie A. et al. Analyzed 16,925 patients experiencing pruritus and found that 17.1% had malignancies, with a notable association with cancers of the liver, gallbladder, biliary tract, hematopoietic system, and skin.^8^ In a similar vein, Kılıç et al. researched 700 patients with solid organ and hematological malignancies, noting that 13% had generalized pruritus together with other skin conditions. Upon investigating the skin findings in patients diagnosed with internal malignancies within the last month, the most frequent conditions identified were tinea pedis/onychomycosis, followed by xerosis (dry skin), and pruritus. These conditions were predominantly observed in patients with hematological malignancies, accounting for 68.96% of cases.^9^ These findings align with these observations, noting a higher prevalence of itching (31.7%) in our cohort, predominantly among colorectal adenocarcinoma patients. This suggests that pruritus may be an important clinical indicator for underlying malignancies, deserving of more rigorous investigation.

### Dermatologic findings and their ımplications

Seborrheic keratoses have been speculated to be potential markers of internal malignancies, particularly in conjunction with acanthosis nigricans. In a study by Fink AM et al., 42% of 150 patients with internal organ malignancies were found to have seborrheic keratoses, with a significant proportion of these patients (62) having gastrointestinal cancers.^10^ The lesions predominantly appeared on the trunk (72.5%) and were least common on the head (5.6%).

These findings indicate a lower occurrence rate of seborrheic keratoses (20.7%) compared to Fink AM et al., yet with a similar distribution, primarily on the trunk. A majority of patients in the present study (90.4%) exhibited a high count of seborrheic keratoses (over 50 lesions), a significant observation given the sparse literature documenting the correlation between the number and placement of these lesions and malignancy.

While the literature lacks comprehensive data on the average age, number, gender distribution, and direct correlation of seborrheic keratosis with BMI, the present study found a higher prevalence of these lesions among patients with a BMI greater than 25, suggesting a noteworthy link that merits further exploration.

These insights enhance our understanding of seborrheic keratosis in the context of internal malignancies and propose that factors like lesion count and BMI might play more critical roles than previously thought. Further research is necessary to elucidate these connections and assess the potential of seborrheic keratoses as indicators of underlying malignancies.

Acanthosis nigricans, known for its links to metabolic issues like obesity, diabetes, and metabolic syndrome, show a highly variable prevalence in literature, ranging from 4.5% to 74%.^11^, ^12^, ^13^, ^14^ This variance may result from different study populations, diagnostic criteria, or associated metabolic conditions.

In comparison to existing studies, our findings indicate a lower prevalence of acanthosis nigricans (10.7%). Bahadursingh S. et al. reported a 52.7% prevalence among 311 patients with type 2 diabetes,^13^ in our cohort, and only 37.5% of patients with acanthosis nigricans had concurrent diabetes. Similarly, the study by Garcia-Hidalgo L. et al.^14^ detected acanthosis nigricans in 29.4% of 156 obese patients, a rate that is notably greater than the 19.2% found in our study of a similar patient population. These disparities could be influenced by ethnic diversity, obesity severity, glycemic control, and diagnostic sensitivity, emphasizing the complex dynamics between acanthosis nigricans and metabolic health.

The present study also detailed the anatomical distribution, most frequently noting the inguinal region, which aligns with the condition's tendency to appear in skin folds. This observation points to a need for further detailed studies on its topography across different demographics.

Moreover, while Brown J. et al.^12^ found a strong association between malignant acanthosis nigricans and gastric carcinoma (55% of cases), our findings indicate a higher link with colorectal cancer (68.7%), followed by gastric adenocarcinoma (18.7%). These discrepancies may be influenced by genetic, environmental, or dietary factors and regional cancer prevalence. The present data, alongside Brown J. et al.'s, underscores the potential of acanthosis nigricans as a paraneoplastic marker, particularly for detecting early gastrointestinal cancers.

These results also corroborate the literature on the association between acquired ichthyosis and gastric adenocarcinoma, with the majority of our cases linked to this type of cancer, reinforcing the observations made by Pérez R. et al. and Saldana M. et al.^15^, ^16^ This recurrent link in the literature and our study suggests that when acquired ichthyosis is diagnosed, especially in the absence of other typical causes, a thorough evaluation for gastric adenocarcinoma should be considered.

The relationship between hypertrichosis lanuginosa and malignancy is similarly intriguing. A review of case reports indicated that out of 64 patients with acquired hypertrichosis lanuginosa, colorectal adenocarcinoma was present in 17 patients and pancreatic adenocarcinoma was noted in one patient.^17^ This pattern of association is mirrored in our study, where 50% of patients with acquired hypertrichosis lanuginosa were found to have colon adenocarcinoma. The consistent association between certain rare dermatologic conditions and specific gastrointestinal malignancies, as observed in our study and supported by literature, suggests a significant, non-random connection with diagnostic implications.

Although the exact mechanisms of these associations remain unclear, the uniformity of findings across studies indicates a biological interaction that deserves further exploration. The present research supports the potential of these dermatologic conditions as markers for gastrointestinal cancers. Clinicians should be aware of these links to enable timely evaluation and management of possible underlying malignancies, potentially improving patient outcomes through early diagnosis.

In clinical examinations, the authors observed that 21.3% of our patients exhibited a widespread presentation of cherry angiomas, with more than 10 lesions each. Factors such as chemical exposure and immunosuppression caused by medications like cyclosporine have been reported to potentially induce or predispose individuals to develop cherry angiomas.^18^ According to Corazza et al., cherry angiomas were found in 15.5% of patients with melanoma and 14.4% of patients with non-melanoma skin malignancies, showing a statistically significant association.^18^ While previous studies have noted a higher incidence of widespread cherry angiomas in older individuals, males, and those with immunosuppression,^18^, ^19^ these findings particularly highlight a significant prevalence in older patients. Further research is needed to determine if widespread cherry angiomas could act as an indicator of malignancy, especially in relation to non-cutaneous malignancies.

### Infections and gastrointestinal malignancies

The present study highlights a significant incidence of fungal pathologies, notably tinea pedis/onychomycosis and oral candidiasis, among individuals with gastrointestinal neoplasms. Notably, the prevalence of onychomycosis in this study group significantly exceeds the global average of approximately 5.5%,^20^ possibly due to factors such as age, sociodemographic variables, reduced self-care, and immunosuppression related to malignancy.

Comparatively, while Perea et al. reported a community prevalence of tinea pedis at 2.9%,^21^ these findings indicate a much higher prevalence of 62% within our cohort, suggesting a marked increase among our subjects. However, the prevalence of tinea cruris in the present study was about 4%, which is consistent with general population rates.

For pityriasis versicolor, our observed incidence rate of 2% in patients with malignancy aligns with the broader range of 0.8% to 5% reported in the literature.^22^, ^23^

Regarding oral candidiasis, which is known to occur in 20% to 40% of individuals undergoing chemotherapy,^24^, ^25^, ^26^ this study found a similar incidence rate of 34.7%. These findings emphasize the increased risk of infectious complications in immunocompromised patients and highlight the need for effective prophylactic and therapeutic strategies to manage fungal infections in this vulnerable group.

Herpes zoster was observed in 6.7% of patients in this study, a rate higher than the general community incidence of 4.8% but lower than the 11.7% prevalence among cancer patients, indicating potential variability influenced by cancer types.^27^ Ongoing research, including a meta-analysis showing a relative risk of 1.42 (95% CI: 1.18, 1.71) for cancer development within one year after a herpes zoster episode, suggests a possible link between herpes zoster and subsequent cancer development.^28^ Furthermore, 5.3% of patients experienced herpes zoster two years prior to their cancer diagnosis, highlighting its potential role as an early indicator of undiagnosed malignancy and emphasizing the need for vigilant clinical assessment in these patients.

### Hair findings

Androgenetic alopecia emerged as the most common hair-related finding in the present study, aligning with its known prevalence in the general population. Anagen effluvium, a rapid and diffuse hair loss triggered by chemotherapy, was another focus of our research. Typically developing within days to weeks after starting cytotoxic treatment, anagen effluvium affects about 65% of patients undergoing chemotherapy, highlighting its significant impact.^29^

Interestingly, this study recorded a lower incidence of anagen effluvium at 25.9%, much less than the commonly reported rate. This notable difference can be attributed to several factors, including the type and dosage of chemotherapeutic agents, the variety of cancers treated, differences in chemotherapy regimens, genetic predispositions, and the use of newer, targeted therapies that tend to have different side effect profiles.

This discrepancy emphasizes the need to consider individual factors in the management and counseling of chemotherapy patients. It shows that while anagen effluvium is a major concern, its severity and likelihood can vary significantly among individuals.

Further studies are needed to better understand the predictors of anagen effluvium and to devise effective strategies to alleviate this distressing side effect. The present research provides additional insights into the frequency and characteristics of anagen effluvium in a specific patient group, potentially aiding in the development of preventive and therapeutic approaches to improve patient quality of life during chemotherapy.

## Conclusion

In conclusion, this study underscores the critical role of dermatological manifestations as potential indicators of gastrointestinal (GI) malignancies, emphasizing the importance of early cancer detection and improved management of skin conditions. By highlighting the correlation between dermatologic symptoms such as pruritus, xerosis, seborrheic keratosis, and acanthosis nigricans, and underlying malignancies, these findings advocate for routine dermatological screening as an integral part of comprehensive patient care. This approach aims to advance early diagnosis and enhance the overall treatment and quality of life for patients with GI cancers, reinforcing the necessity of dermatological assessment in oncology protocols.

## Financial support

None declared.

## Authors’ contributions

Burcu Aydemir Demirci: Concept; design; supervising; financing and equipment;data collection and entry; analysis and interpretation; literature search; writing.

Deniz Aksu Arıca: Concept; design; supervising; financing and equipment; data collection and entry; analysis and interpretation; literature search; writing; critical review.

Leyla Baykal Selcuk: Financing and equipment; data collection and entry; analysis and interpretation; literature search; critical review.

Evren Fidan: Supervising; financing and equipment; data collection and entry; analysis and interpretation.

Ali Güner: Concept; design; supervising; financing and equipment; data collection and entry; analysis and interpretation; writing; critical review.

Savaş Yaylı: Supervising.

İbrahim Etem Arıca: Design; literature search.

## Conflicts of interest

None declared.
